# Biology and Management of Dedifferentiated Liposarcoma: State of the Art and Perspectives

**DOI:** 10.3390/jcm10153230

**Published:** 2021-07-22

**Authors:** Jun Nishio, Shizuhide Nakayama, Kazuki Nabeshima, Takuaki Yamamoto

**Affiliations:** 1 Department of Orthopaedic Surgery, Faculty of Medicine, Fukuoka University, 7-45-1 Nanakuma, Jonan-ku, Fukuoka 814-0180, Japan; n.shizuhide@gmail.com (S.N.); yamamotot@fukuoka-u.ac.jp (T.Y.); 2 Department of Pathology, Faculty of Medicine, Fukuoka University, 7-45-1 Nanakuma, Jonan-ku, Fukuoka 814-0180, Japan; kaznabes@fukuoka-u.ac.jp

**Keywords:** dedifferentiated liposarcoma, well-differentiated liposarcoma, atypical lipomatous tumor, diagnosis, pathogenesis, treatment

## Abstract

Dedifferentiated liposarcoma (DDL) is defined as the transition from well-differentiated liposarcoma (WDL)/atypical lipomatous tumor (ALT) to non-lipogenic sarcoma, which arises mostly in the retroperitoneum and deep soft tissue of proximal extremities. It is characterized by a supernumerary ring and giant marker chromosomes, both of which contain amplified sequences of 12q13-15 including *murine* *double minute 2* (*MDM2*) and *cyclin-dependent kinase 4* (*CDK4*) cell cycle oncogenes. Detection of *MDM2* (and/or *CDK4*) amplification serves to distinguish DDL from other undifferentiated sarcomas. Recently, *CTDSP1/2*-*DNM3OS* fusion genes have been identified in a subset of DDL. However, the genetic events associated with dedifferentiation of WDL/ALT remain to be clarified. The standard treatment for localized DDL is surgery, with or without radiotherapy. In advanced disease, the standard first-line therapy is an anthracycline-based regimen, with either single-agent anthracycline or anthracycline in combination with the alkylating agent ifosfamide. Unfortunately, this regimen has not necessarily led to a satisfactory clinical outcome. Recent advances in the understanding of the pathogenesis of DDL may allow for the development of more-effective innovative therapeutic strategies. This review provides an overview of the current knowledge on the clinical presentation, pathogenesis, histopathology and treatment of DDL.

## 1. Introduction

Adipocytic tumors are frequently encountered in routine practice. The 2020 World Health Organization Classification of Tumors of Soft Tissue and Bone recognizes five major liposarcoma subtypes: well-differentiated liposarcoma (WDL)/atypical lipomatous tumor (ALT); dedifferentiated liposarcoma (DDL); myxoid liposarcoma; pleomorphic liposarcoma; and myxoid pleomorphic liposarcoma [1]. DDL is characterized as a typically non-lipogenic sarcoma that is juxtaposed to WDL/ALT. Dedifferentiation occurs in up to 10% of WDL/ALT cases [2]. The incidence of DDL is less than 0.1 per 1,000,000 each year [3]. DDL genetically overlaps with WDL/ALT; both entities are associated with high level amplifications of *murine* *double minute 2* (*MDM2*) and *cyclin-dependent kinase 4* (*CDK4*) cell cycle oncogenes within 12q13-15. DDL also shows recurrent amplifications of 1p32 and 6q23 [2]. Recently, novel fusion genes involving *DNM3 opposite strand/antisense RNA* (*DNM3OS*) have been identified in a subset of DDL [3]. Surgery remains the mainstay of treatment for localized DDL. Systemic treatment with chemotherapy and molecular targeted agents is one of the main therapeutic modalities in patients with advanced or metastatic disease. In this article, we review the key clinical, histopathological and genomic characteristics of DDL, summarize the current management and provide an overview of the ongoing research of novel therapeutic strategies.

## 2. Clinical Characteristics

DDL presents most frequently in middle-aged and older adults (peak incidence in the sixth to seventh decades). It is a rare neoplasm in children and young adults [4]. There is a no gender predilection. Retroperitoneum is the most common location and DDL is the most frequent retroperitoneal sarcoma. DDL can also occur in the extremities, spermatic cord, trunk (including mediastinum and thorax) and head and neck [1]. In our experience, occurrence in the superficial soft tissue is extremely rare.

DDL usually presents as a large painless mass, often with a history of several years of slow enlargement [5]. In the retroperitoneum, it may be detected incidentally during radiological imaging. Presenting symptoms are typically related to the location of origin. Dedifferentiation is likely a time-dependent phenomenon and up to 90% of DDL cases arise de novo [1]. In the remaining 10% of cases, DDL develops as a dedifferentiated recurrence of a previous WDL/ALT.

Unlike WDL/ALT, DDL is a high-grade and aggressive disease, with a local recurrence rate of approximately 40%, metastatic rate of 15-30% and overall mortality rate of 28% [6,7]. The most important prognostic factor for DDL is anatomical location. Actually, a few studies showed that the extremities were a favorable location for DDL compared with the retroperitoneum [8,9].

## 3. Imaging Features

### 3.1. MRI

**Figure 1:**
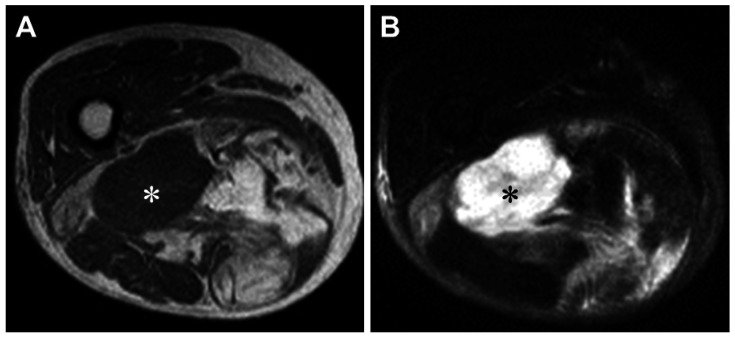
Magnetic resonance imaging of dedifferentiated liposarcoma in the right thigh of an 83-year-old woman. Axial T1-weighted (**A**) and T2-weighted spectral presaturation with inversion recovery (**B**) sequences display a large soft tissue mass composed of non-lipomatous (white and black asterisks) and juxtaposed lipomatous components.

Magnetic resonance imaging (MRI) is the preferred modality for evaluating soft tissue lesions and is helpful in demonstrating the fatty nature of the tumor. DDL represents a biphasic neoplasm, with one component being a WDL/ALT and the other a non-lipogenic sarcoma (Figure 1). The WDL/ALT component demonstrates high signal intensity on both T1- and T2-weighted images, consistent with a lipomatous tumor. The dedifferentiated component is usually larger than 3 cm [10] and typically shows a non-specific MR appearance with prolonged T1 and T2 relaxation times. Hemorrhage and necrosis may be seen within the high-grade dedifferentiated component. In our limited experience, gadolinium contrast enhancement of the dedifferentiated component is variable.

### 3.2. 18F-FDG PET/CT

Positron emission tomography (PET) is the gold standard in metabolic imaging. The radionuclide most commonly used for PET is fluorodeoxyglucose (FDG). High-grade malignancies tend to have higher rates of glycolysis and FDG uptake than those of intermediate malignancies and benign lesions. The dedifferentiated component displays high FDG uptake, whereas the WDL/ALT component shows almost no FDG uptake [8]. It is recognized that PET/computed tomography (CT) would be useful for identifying the presence of dedifferentiation within the tumor. Moreover, PET/CT can be helpful in guiding the location for biopsy in this heterogeneous tumor [11,12].

## 4. Pathogenesis

**Figure 2:**
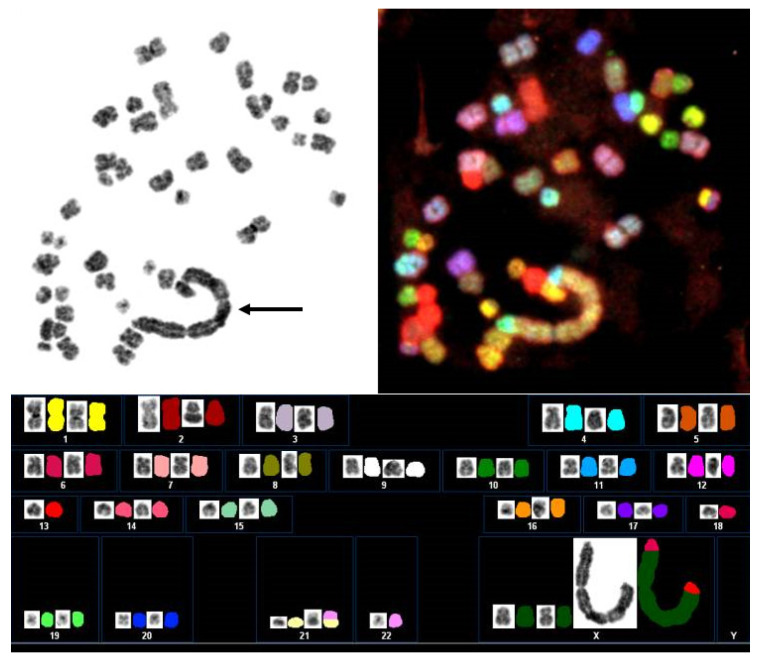
Giant marker chromosome in dedifferentiated liposarcoma. Spectral karyotyping demonstrates that the giant marker (arrow) is mainly composed of material from the X chromosome.

Karyotypes and quantitative genomic profiles of DDL are often more complex than those of WDL/ALT. DDL is cytogenetically characterized by a supernumerary ring and giant marker chromosomes [1,13,14,15]. These rings and giant markers contain amplified sequences of 12q13-15 and other co-amplified chromosomal regions (Figure 2). The 12q13-15 region includes a number of genes such as *MDM2*, *CDK4*, *high mobility group AT-hook 2* (*HMGA2*), *tetraspanin 31* (*TSPAN31*), *YEATS domain containing 4* (*YEATS4*), *carboxypeptidase M* (*CPM*) and *solute carrier family 35 member E3* (*SLC35E3*) [3,5]. *MDM2* is the main driver gene with the 12q amplicon. MDM2 binds to p53 and negatively regulates it by preventing nuclear translocation and transcription and by promoting its degradation via an E3 ubiquitin ligase [16]. *CDK4* encodes a 33-kD protein that is a key factor in the regulation of the G1-S translation of the cell cycle. Accumulation of the CDK4-CCDN1 complex leads to phosphorylation of the retinoblastoma (RB) protein [14]. In current practice, immunohistochemistry for MDM2 and CDK4 can be helpful to screen for 12q13-15 amplification. *HMGA2* encodes a protein in the nonhistone chromosomal high-mobility group (HMG) protein family that contains DNA-binding domains and can act as a transcriptional regulating factor. *HMGA2* and *TSPAN31* are commonly coamplified with *MDM2*, implicating a critical role in the development of DDL [17,18]. *YEATS4* encodes a putative transcription factor required for physiologic suppression of p53 function and its knockdown reduces DDL cell proliferation [19]. *CPM* belongs to the family of the carboxypeptidases and its knockdown results in the inhibition of DDL cell growth, migration and invasion [20]. *YEATS4* and *CPM*, frequently coamplified with *MDM2*, are known to be involved in the dedifferentiation process [21]. *SLC35E3* is a protein coding gene and the simultaneous gain of *MDM2*, *CPM* and *SLC35E3* is likely a crucial step during the development of DDL [3].

In addition to the 12q13-15 amplification, high-level amplifications of 1p32 and 6q23 are found in DDL and are associated with a worse prognosis [22]. It is of great interest that coamplifications of 1p32 and 6q23 are mutually exclusive and never seen in WDL. *Jun proto-oncogene* (*JUN*) and *mitogen-activated protein kinase kinase kinase 5* (*MAP3K5*) are upregulated through amplifications in 1p32 and 6q23, respectively [22]. *JUN* encodes part of the activator protein transcription factor (AP-1) complex involved in cell proliferation, transformation and apoptosis and inhibits peroxisome proliferator-activated receptor gamma (PPARγ), a key mediator of adipocytic differentiation [14,23]. *MAP3K5* encodes a MAP3 kinase involved in the Jun N-terminal kinase (JNK) signaling pathway [14,24]. *MAP3K5* amplification activates JNK ultimately leading to JUN activation and PPARγ inactivation. It is therefore suggested that amplifications of *JUN* and/or *MAP3K5* may directly block adipocytic differentiation in DDL. Saâda-Bouzid et al. reported that CDK4 and JUN amplification were associated with a poor outcome. Moreover, receptor tyrosine kinase (RTK) genes are also amplified in DDL, including *discoidin domain receptor tyrosine kinase 2* (*DDR2*), *Erb-B2 receptor tyrosine kinase 3* (*ERBB3*), *neurotrophic tyrosine receptor kinase 1* (*NTRAK1*), *fibroblast growth factor receptor 1* (*FGFR3*) and *ROS Proto-Oncogene 1* (*ROS1*) [25]. Asano et al. suggested that amplification of these RTK genes is probably involved in DDL progression [25]. Based on these findings, we speculate that tyrosine kinase inhibitors (TKIs) may be an effective therapeutic option for DDL patients with RTK gene amplification. On the other hand, somatic point mutations are uncommon in DDL [26].

A recent study on the integrated exome and RNA sequencing of DDL demonstrated *CTD small phosphatase 1* (*CTDSP1*)-*DNM3OS* and *CTD small phosphatase 2* (*CTDSP2*)-*DNM3OS* as recurrent fusion genes [3]. *CTDSP1* and *CTDSP2* encode the C-terminal domain small phosphatases 1 and 2 and knockdown of *CTDSP2* reduces DDL cell proliferation [20]. *DNM3OS* is located at 1q24.3 and encodes the microRNA cluster miR-199a∼214 [27]. Interestingly, DDL with these fusion genes showed the significant upregulation of *DNM3OS* and the gain of 1q24.3 were associated with poor progression-free survival (PFS) [3]. These findings suggest that upregulation of *DNM3OS* may contribute to DDL progression.

## 5. Histopathology

The histological hallmark of DDL is transition from WDL/ALT to non-lipogenic sarcoma [1]. However, it may be difficult to identify the WDL/ALT component in some cases. In current practice, fluorescence in situ hybridization (FISH) for the assessment of *MDM2* amplification status can serve as a useful diagnostic adjunct for DDL.

**Figure 3:**
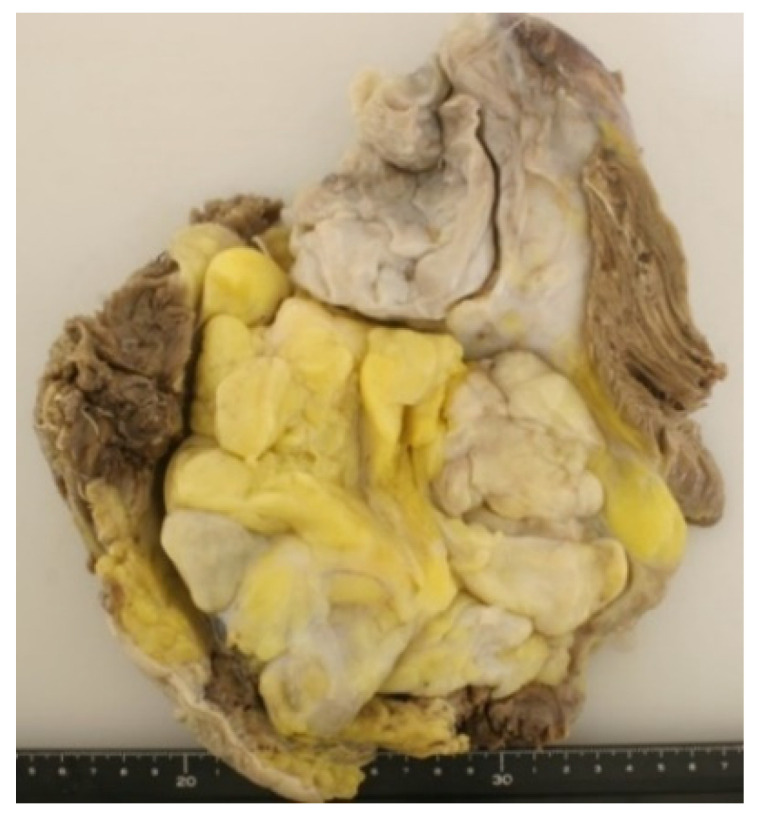
Cut surface showing a multilobulated appearance with gray-white and yellow areas.

Grossly, DDL usually appears as a large (>10 cm) multinodular yellow mass containing a discrete, solid, often freshy non-lipomatous area [1]. The cut surface is variable, ranging from gray-white to tan (Figure 3). Necrosis or hemorrhage may be seen within the dedifferentiated area.

**Figure 4:**
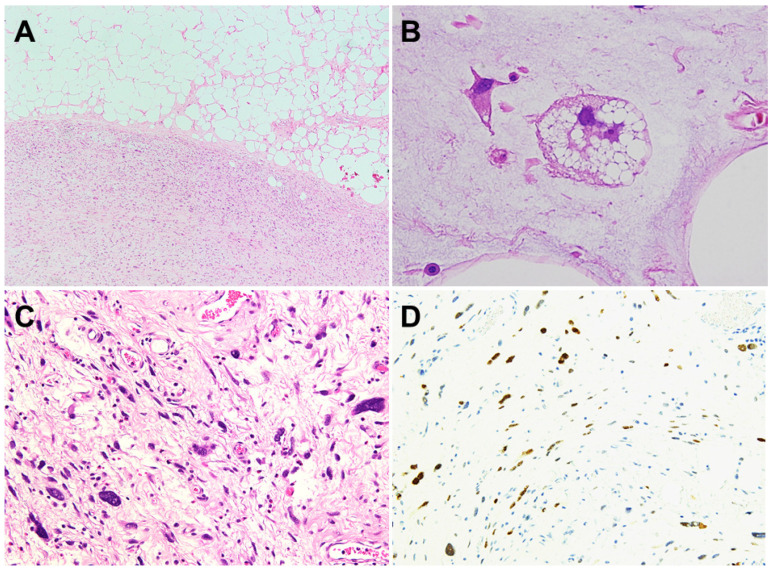
Histopathology of dedifferentiated liposarcoma. (**A**) Abrupt transition from well-differentiated liposarcoma (WDL)/atypical lipomatous tumor (ALT) to high-grade non-lipogenic sarcoma is seen. (**B**) Multivacuolated lipoblast can be seen in the WDL/ALT area. (**C**) The dedifferentiated component consists of atypical spindle cells, round to polygonal cells and bizarre giant cells, resembling myxofibrosarcoma. (**D**) MDM2 expression in the dedifferentiated area.

Histologically, DDL usually show an abrupt transition between well-differentiated and dedifferentiated areas (Figure 4A). Well-dedifferentiated areas consist of mature fat cells with a significant variation in size and atypical, hyperchromatic stromal spindle cells. A varying number of monovacuolated or multivacuolated lipoblasts may be seen (Figure 4B). The extent of dedifferentiation is variable. Dedifferentiated areas exhibit a wide morphological spectrum but most frequently resemble undifferentiated pleomorphic sarcoma or high-grade myxofibrosarcoma (Figure 4C). The mitotic activity is variable and usually lower than that seen in other high-grade sarcomas. The concept of low-grade dedifferentiation is now widely recognized [6]. Low-grade dedifferentiation is characterized by the presence of bland fibroblast-like spindle cells with mild nuclear atypia and low mitotic activity [1]. In approximately 5–10% of cases, DDL undergoes heterogeneous differentiation [1]. The most frequent lines of dedifferentiation include myogenic, osteosarcomatous or chondrosarcomatous elements [6,28,29,30]. Myogenic dedifferentiation encompasses rhabdomyosarcomatous or leiomyosarcomatous elements. It is of interest that myogenic dedifferentiation, particularly with rhabdomyosarcomatous elements, is associated with a significantly worse outcome [28,29]. Rarely, a peculiar neural-like or meningothelial-like whirling pattern has been described in association with metaplastic ossification [31,32]. It is now recognized that most neoplasms previously diagnosed as inflammatory malignant fibrous histiocytoma or myxofibrosarcoma in the retroperitoneum represent DDL [7].

The only reliable marker is the consistent nuclear reactivity of MDM2 (Figure 4D) and CDK4 [33]. In our experience, MDM2 and CDK4 expression is usually prominent in the dedifferentiated area compared to the WDL/ALT area. In the differential diagnosis of DDL, p16 had good sensitivity (94.4%) but lower specificity (70%) [34]. Although the use of p16 as a single immunohistochemical marker is limited due to its specificity, the combination of MDM2, CDK4 and p16 may be helpful in distinguishing DDL from other adipocytic neoplasms including pleomorphic liposarcoma [35]. DDL can exhibit variable expression of CD34 [36]. The S100 protein is absent in non-lipogenic areas of DDL.

**Figure 5:**
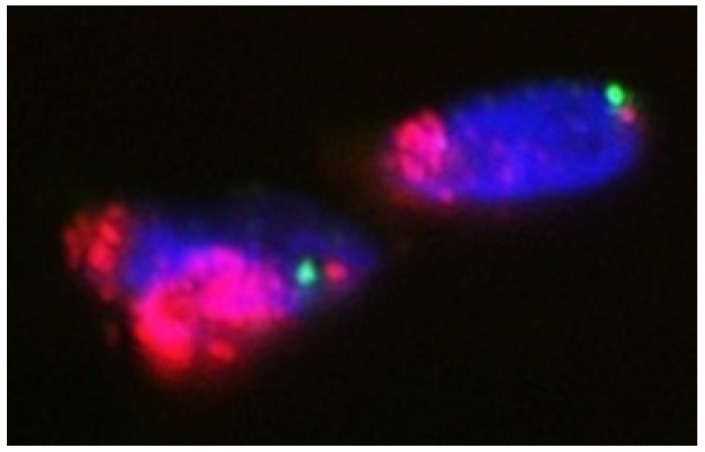
Interphase fluorescence in situ hybridization analysis using probes for *MDM2* (red signals) and centromere of chromosome 12 (green signals) showing high-level amplification of *MDM2*.

In our experience, FISH is a useful adjunct in the diagnosis of DDL, especially when a corresponding WDL/ALT component is absent or obscure (Figure 5). There are several studies that evaluated the utility of *MDM2* amplification in DDL. Weaver et al. reported 100% sensitivity and specificity for *MDM2* amplification in distinguishing between benign lipomatous tumors and DDL [37]. Kimura et al. showed 100% sensitivity and 95% specificity for *MDM2* amplification in distinguishing DDL from other spindle and pleomorphic sarcomas [38]. The detection of *CDK4* amplification by FISH is also helpful for distinguishing DDL from its histological mimics in the appropriate clinical context [14,15,39]. There are several studies that investigated the prognostic significance of these molecular alterations in DDL. Italiano et al. demonstrated that DDL with *MDM2* amplification but no *CDK4* amplification had a favorable prognosis [40]. Jour et al. reported that *MDM2* amplification level was not a significant prognostic factor [41]. In that study, the authors suggested that Fédération Nationale des Centres de Lutte Contre le Cancer (FNCLCC) grading of DDL may predict a greater risk of local recurrence in FNCLCC grade 3 tumors. In contrast, Ricciotti et al. found that high *MDM2* and *CDK4* amplification levels (>38 and >30 copies, respectively) were associated with worse disease-free survival and disease-specific survival [42]. Similarly, Lee et al. showed that high-level amplification of *CDK4* was a poor prognostic marker [43].

## 6. Management

### 6.1. Localized Disease

Wide resection is the standard treatment for local disease. Resection with R0 margin is achievable for DDL located in the extremities but is more challenging for retroperitoneal tumors. In surgical practice, selection of which procedure is suitable for an individual patient must be based on tumor location, size, stage, relationship with surrounding neurovascular and bone elements and functional and cosmetic requirements.

Although some authors described improved local control associated with the use of radiation therapy (RT) in addition to surgery [44,45,46], a recent phase 3, randomized European Organization for Research and Treatment of Cancer (EORTC)-62092 trial showed that preoperative RT should not be considered as standard of care for primary retroperitoneal DDL [47]. On the other hand, the utility of RT for local control of primary extremity DDL remains unclear [8,9] and there is controversy regarding its optimal timing. We now use RT when the resection proves R1/R2 in extremity tumors close to major nerves and vessels.

### 6.2. Advanced Disease

The development of unresectable local and/or metastatic DDL is associated with a poor prognosis. In this section, we summarize an update of the current management of advanced DDL and highlight ongoing and future research.

#### 6.2.1. Anthracycline-Based Therapy

As with other soft tissue sarcoma (STS) subtypes, anthracycline-based therapy is a standard first-line treatment for advanced DDL [48,49]. Recent randomized phase 3 trials failed to demonstrate an improvement in overall survival (OS) with the addition of ifosfamide or other types of alkylator agents to doxorubicin in patients with metastatic STS [50,51,52]. In the EORTC-62012 phase 3 trial, post hoc subgroup analysis showed no improvement in objective response rate (ORR) or OS in patients with liposarcoma treated with combination therapy with doxorubicin and ifosfamide compared to doxorubicin alone [53]. In this trial, it was not specified how many had DDL.

There are several retrospective studies regarding the role of anthracycline-based treatment in patients with advanced DDL [54,55,56]. In the largest multi-institutional study, of the 208 patients, 171 (82%) had DDL. Approximately 82% of patients received anthracycline-based therapy. Among 167 evaluable patients, objective response (OR) was observed in 21 patients (12%). Median PFS was 4.6 months and median OS was 15.2 months [55]. The largest single-center study of 82 patients with DDL treated with first-line chemotherapy showed that median PFS in the advanced setting was 4 months and median OS from the start of chemotherapy was 29 months. Among 51 evaluable patients treated in unresectable/metastatic setting, OR was observed in 10 patients (20%) [56]. Taken together, it is reasonable to recommend that anthracycline-based regimens can be considered as a front-line treatment for advanced DDL.

#### 6.2.2. Pazopanib

Pazopanib is an oral multi-target TKI with anti-angiogenic and antitumorigenic properties and has been approved in multiple countries as a second or later line treatment for patients with advanced STS. A single-arm phase 2 trial revealed that pazopanib was inactive in the liposarcoma subgroup [57]. In this EORTC-62043 trial, only 3 (17.6%) of 17 patients met the criteria for a positive response. A subsequent randomized double-blind multicenter phase 3 trial excluded liposarcomas based on the EORTC-62043 data [58]. However, following a centralized histopathological review, 5 (26.3%) of the 19 patients with liposarcoma had a progression-free rate (PFR) at 12 weeks, which would have met the threshold for further investigation in this study [57]. In recent years, a prospective single-arm multicenter phase 2 trial was performed to support the efficacy of pazopanib for advanced liposarcoma [59]. In this NCT01506596 trial, of the 41 patients, 27 (65.9%) had DDL. Median PFS for patients with DDL was 6.24 months. Median OS among all patients was 12.6 months. Another phase 2 clinical trial (NCT01692496) was designed to assess PFS at 12 weeks and was achieved in 43.2% of patients with advanced WDL/DDL [60]. In the WDL/DDL subgroup, median PFS and OS were 3.5 and 16.4 months, respectively. In a randomized phase 2 clinical trial (EPAZ) assigned to doxorubicin or pazopanib, for both PFS and OS, pazopanib showed non-inferiority compared to doxorubicin with similar quality of life measure outcome [61]. These phase 2 studies suggested that the use of pazopanib in treating advanced DDL may show promise [59,60,61]. More recently, Suehara et al. indicated that pazopanib is possibly a favorable clinical option in advanced STS with *GLI Family Zinc Finger 1* (*GLI*) amplification such as DDL [62]. The role of pazopanib in advanced DDL remains unclear and requires further investigation in a phase 3 study.

Other multi-target TKIs such as sunitinib [63], regorafenib [64] and anlotinib [65] have also been investigated in phase 2 trials in advanced STS including liposarcoma. None are currently licensed for use in liposarcoma.

#### 6.2.3. Eribulin

Eribulin, a non-taxane microtubule dynamics inhibitor, is currently licensed for use in patients with unresectable or metastatic liposarcoma who received a prior anthracycline-based regimen. The approval was based on results from a randomized open-label multicenter phase 3 trial enrolling 452 patients with advanced liposarcoma or leiomyosarcoma [66]. In this E7389-G000-309 trial, OS was significantly improved in the eribulin arm compared to the dacarbazine arm (median OS, 13.5 versus 11.5 months; hazard ratio (HR) 0.77; 95% confidence interval (CI) 0.62–0.95; *p* = 0.0169), despite there being no significant difference between the two arms in median PFS. Subsequently, a subgroup analysis by histological subtype, using data from the E7389-G000-309 study, indicated that among the total of 65 patients with DDL, median OS was 18.0 months in those receiving eribulin compared to 8.1 months in those receiving dacarbazine [67]. Therefore, eribulin can be expected to improve OS in patients with advanced DDL.

#### 6.2.4. Trabectedin

Trabectedin, a marine-derived drug that binds to the minor groove of DNA, has also been approved by the United States Food and Drug Administration (FDA) and European Medicines Agency (EMA) for treatment of patients with unresectable or metastatic liposarcoma who received a prior anthracycline-based regimen. The approval was based on results from a randomized open-label multicenter phase 3 trial enrolling 518 patients with advanced liposarcoma or leiomyosarcoma [68]. In this ET743-SAR-3007 trial, PFS was significantly improved in the trabectedin arm compared to the dacarbazine arm (median PFS, 4.2 versus 1.5 months; HR 0.55; 95% CI 0.44–0.70; *p* < 0.001). However, the final OS analysis demonstrated no significant improvement in OS of trabectedin over dacarbazine [69]. A subgroup analysis by histological subtype, using data from the ET743-SAR-3007 study, showed that among the total of 70 patients with DDL, median PFS was 2.2 months in those receiving trabectedin compared to 1.9 months in those receiving dacarbazine [68]. In the retrospective case series, Fabbroni et al. suggested that trabectedin may be more active against WDL/low-grade DDL than in high-grade DDL [70].

#### 6.2.5. Gemcitabine and Docetaxel

The combination of gemcitabine and docetaxel has activity in patients with advanced STS [71]. A subgroup analysis by histological subtype, using data from a randomized open-label phase 2 study (NCT00142571), revealed that the use of gemcitabine and docetaxel or gemcitabine alone demonstrated stable disease (SD) in 9 (75%) of the 12 patients with advanced WDL/DDL, although most responses were for less 24 weeks [72]. A randomized controlled phase 3 trail (GeDDiS) was performed to compare the efficacy of gemcitabine and docetaxel versus doxorubicin in the first-line setting for advanced STS [73]. There was no significant difference between the two arms in PFS and OS, which contained eight and five patients with DDL, respectively. The precise role of gemcitabine and docetaxel in advanced DDL remains to be defined, particularly the potential for combination therapy.

#### 6.2.6. MDM2-Thargeted Therapy

MDM2 is a critical component of DDL tumorigenesis [74]. An exploratory proof-of-mechanism study demonstrated adequate safety, tolerability, p53 activation, antiproliferative activity and preliminary antitumor efficacy of the investigational MDM2 inhibitor RG7112 in patients with operable *MDM2*-amplified WDL/DDL [75]. A first-in-human phase 1 trial (NCT01636479) of SAR405838, an oral spirooxindole inhibitor of MDM2, showed no OR; however, SD was observed in 22 (71%) of the 31 patients with DDL [76]. Progression-free response (PFR) at 3 months was met by 32% of patients [76]. Another phase 1 trial of MK-8242, a small molecule inhibitor of MDM2, showed that ORR was 11.1% in the 27 patients with advanced WDL/DDL and median PFS was 5.5 months in the 16 patients with advanced DDL [77].

Additional MDM2 inhibitors of several other classes are under ongoing investigation, including milademetan (DS-3032b), idasanutlin (RG7338), CPM097, ALRN-6924 and JNJ-26854105. Updated results from a phase 1 study (NCT01877382) of milademetan enrolling 40 patients with WDL/DDL reported preliminary clinical activity and an acceptable safety profile [78]. A partial response (PR) was seen in one patient with DDL [78]. Although *TP53* mutations appear in circulating cell-free DNA of patients with DDL during treatment with the MDM2 inhibitor [79], targeting MDM2 is a promising treatment strategy for this disease.

#### 6.2.7. CDK4-Thageted Therapy

*CDK4* is amplified in over 90% of DDL [49]. Three CDK4/6 inhibitors are currently approved in clinical practice, namely: palbociclib, ribociclib and abemaciclib. Palbociclib, a potent oral inhibitor of CDK4 and CDK6, induces cell cycle arrest in CDK4-amplified WDL/DDL cells [19]. In a non-randomized open-label phase 2 study (NCT01209598), of the 60 patients, 47 (78%) had DDL and received palbociclib at 125 mg daily for 21 days in 28-day cycles [80]. Median PFS was 17.9 weeks, with overall PFS at 12 weeks of 57.2% and a manageable toxicity profile. One patient achieved a complete response (CR) [80].

Several other CDK4/6 inhibitors are under ongoing investigation, including ribociclib [81] and abemaciclib [82]. In an open-label phase 1 study (NCT01237236) of single-agent ribociclib in liposarcoma, no OR was observed (0 of 39 evaluable patients), although SD surpassing 6 months was seen in 6 patients (15%) [81]. In a single-arm phase 2 study (NCT02846987), 30 patients with advanced DDL (29 evaluable for response) were enrolled to receive oral abemaciclib at 200 mg twice daily without interruption. Median PFS was 30.4 weeks, with overall PFS at 12 weeks of 76% and a manageable toxicity profile. There was one PR and a further 3 patients had a reduction of more than 10% in tumor size by the Response Evaluation Criteria in Solid Tumors (RECIST) guidelines (version 1.1) [82].

The palbociclib and RG7388 (MDM2 inhibitor) combination in DDL led to an increased rate of apoptosis, recued tumor growth and had a significant increase in median PFS compared to a single agent alone [83]. A phase 1b study (NCT02343172) assessing the safety and efficacy of HDM201 (MDM2 inhibitor) in combination with LEE001 (ribociclib) is currently ongoing in patients with advanced DDL.

#### 6.2.8. Exportin 1 (XPO1) Inhibitor

Nuclear export is a rational target in DDL [49]. XPO1 mediates nuclear export of multiple tumor suppressor and growth regulatory proteins. XPO1 is highly expressed in different histological subtypes of liposarcoma including DDL [84]. Selinexor, an oral selective inhibitor of XPO1, significantly inhibited cellular proliferation and induced cell cycle arrest and apoptosis of DDL both in vitro and in vivo [84]. Recently, Zuco et al. demonstrated that antitumor activity of selinexor was higher than doxorubicin in DDL patient-derived xenografts and cell lines [85]. In a phase 1b clinical trial (NCT01896505), although no OR was observed, selinexor demonstrated antitumor activity in patients with advanced DDL, showing a reduction in the target lesion size from baseline in 6 (40%) of 15 patients, with 47% of patients experiencing a best response of SD for at least 4 months [86]. A subsequent randomized double-blinded placebo-controlled multicenter phase 2/3 (SEAL) study (NCT02606461) was initiated to assess the efficacy, safety and health-related quality of life (HRQoL) of patients with advanced DDL treated with either selinexor or placebo. This phase 3 SEAL study demonstrated enhanced clinical activity and a manageable safety profile in patients with DDL compared to placebo [87]. Median PFS was 2.83 months in the selinexor arm versus 2.07 months in the placebo arm (HR 0.70; *p* = 0.0228). There was no significant difference between the two arms in OS [87]. Most recently, Gounder et al. reported that pain scores worsened in the placebo arm compared to the selinexor arm across all postbaseline visits, although some visits (day 43 and 85) were not statistically significant [88].

#### 6.2.9. Immunotherapy

The major targets of FDA-approved immunotherapeutic antibodies are programmed cell death protein-1 (PD-1), its ligand programmed cell death ligand-1 (PD-L1) and cytotoxic T lymphocyte-associated antigen-4 (CTLA-4) [89]. PD-1 is normally expressed on the surface of activated T cells and suppresses unwanted or excessive immune responses. PD-L1 is widely expressed in a variety of cells. CTLA-4 is a protein receptor expressed on the T lymphocyte surface that plays a crucial role during T cell activation. The PD-1/PD-L1 interaction is a major pathway hijacked by tumors to suppress immune control. Several studies have assessed the expression of PD-L1 in DDL [90,91,92]. PD-L1 positive expression (≥1%) was identified in 21.9% (7/32) of the DDL cases [90]. The ≥1% PD-L1 expression group demonstrated a significantly worse recurrence-free survival (RFS) (*p* = 0.027) and OS (*p* = 0.017) compared to the no PD-L1 expression group [90]. Miyake et al. also reported that DDL showed a significantly higher level of PD-L1 expression (*p* = 0.02) compared to other STSs [92]. These studies suggest the possibility of this pathway-targeted immunotherapy for advanced DDL.

Pembrolizumab and nivolumab are the two most representative PD-1 inhibitors. In a single-arm open-label multicenter phase 2 (SARC028) trial (NCT02301039), pembrolizumab demonstrated promising activity in patients with advanced DDL [93]. Among 10 evaluable patients with DDL, 2 (20%) had PR and 4 (40%) had SD. Median PFS was 25 weeks and PFS rate was 60% at 12 weeks [93]. The promising clinical results of the SARC028 study led to the enrollment of an expansion cohort consisting of an additional 30 DDL/pleomorphic liposarcoma patients. In the liposarcoma cohort, ORR was 10% (4 of 39 patients with PR) and PFS rate was 44% at 12 weeks. Median PFS was 2 months and median OS was 13 months [94]. In a randomized open-label non-comparative multicenter phase 2 (Alliance A091401) trial (NCT02500797), patients with advanced/metastatic sarcoma received either nivolumab alone or nivolumab in combination with ipilimumab (CTLA-4 inhibitor) [95]. ORR was 5% in the nivolumab monotherapy group and 16% in the combination group. Median PFS was 1.7 and 4.1 months, respectively. Median OS was 10.7 and 14.3 months, respectively [95]. The promising results of the Alliance A091401 study led to the enrollment of an expansion cohort consisting of an additional 24 DDL patients. In the DDL cohort, the primary end point of 6-month response rate was met with the combination nivolumab plus ipilimumab but not with nivolumab alone. ORR was 6.7% in the nivolumab monotherapy group and 14.3% in the combination group. Median PFS was 4.6 and 5.5 months, respectively. Median OS was 8.1 and 13.1 months, respectively [96]. Additionally, a randomized phase 2 clinical trial (NCT03307616) evaluating the efficacy of neoadjuvant checkpoint blockade (nivolumab or nivolumab/ipilimumab) in patients with surgically resectable primary or recurrent retroperitoneal DDL is currently under way [97]. Preliminary results showed that median pathological response was 22.5% and median change in tumor size (radiological response) was +9% in the DDL cohort [98].

The combination of immunotherapy and RT may have the potential to elicit a systemic immune response to improve long-term survival in patients with advanced DDL. A randomized controlled phase 2 (SU2C-SARC032) trial (NCT03092323) to evaluate the safety and efficacy of neoadjuvant pembrolizumab with concurrent RT and adjuvant pembrolizumab compared to neoadjuvant RT alone in patients with high-risk extremity STS including DDL is currently ongoing [99].

## 7. Conclusions

DDL typically arises in the retroperitoneum or proximal extremities of middle-aged and older adults and is defined as the transition from WDL/ALT to non-lipogenic sarcoma, either in the primary tumor or in a recurrence of WDL/ALT. It is cytogenetically characterized by a supernumerary ring and giant marker chromosomes. These rings and giant markers contain amplified sequences of 12q13-15 including *MDM2* and *CDK4*. In addition, the *CTDSP1/2*-*DNM3OS* fusion genes have been identified in a subset of DDL. The detection of *MDM2* (and/or *CDK4*) amplification by FISH is a useful ancillary tool in the diagnosis of DDL. Surgical resection is the mainstay of treatment for localized DDL, although the use of RT or systemic therapies in conjunction with surgery may be considered in very selected patients. Anthracycline-based therapy is a standard first-line treatment for advanced DDL. Eribulin and trabectedin are currently the two most promising and evidenced-based second-line treatment options for advanced DDL. Pazopanib is possibly a favorable clinical option in advanced *GLI*-amplified DDL. MDM2 and CDK4 inhibitors have shown some evidence of efficacy in advanced DDL. However, the precise role of these agents remains to be elucidated, particularly the potential for combination therapy. Several other promising agents are currently under investigation for the treatment of advanced DDL in phase 2/3 clinical trials, including XPO1 and PD-1 inhibitors. In the future, we expect that a wide range of treatment options will be available to patients with this disease.
